# The Immunomodulatory Effect of β-Glucan Depends on the Composition of the Gut Microbiota

**DOI:** 10.3390/foods12173148

**Published:** 2023-08-22

**Authors:** Miseon Sung, Yohan Yoon, Jeeyeon Lee

**Affiliations:** 1 Department of Food and Nutrition, Sookmyung Women’s University, Seoul 04310, Republic of Korea; koof97@naver.com (M.S.); yyoon@sookmyung.ac.kr (Y.Y.); 2 Risk Analysis Research Center, Sookmyung Women’s University, Seoul 04310, Republic of Korea; 3 Department of Food & Nutrition, Dong-eui University, Busan 47340, Republic of Korea

**Keywords:** β-glucan, immunomodulatory effect, gut microbiota composition, health-functional food

## Abstract

This study aimed to elucidate the relationship between the immunomodulatory effects of β-glucan and the composition of gut microbiota in mice. The mice were fed a diet containing β-glucan for 3 weeks, and feces, blood, and tissues were then collected to analyze the immunomodulatory effect and gut microbiota composition. Based on the results of the analysis of the expression level of immune-associated proteins, the high immunomodulatory effect group (HIE) and low immunomodulatory effect group (LIE) were categorized. Before the β-glucan diet, the proportions of the phylum Bacteroidota, family *Muribaculaceae*, and family *Lactobacillaceae* were significantly higher in HIE than in LIE. Furthermore, the genus *Akkermansia* was absent before the β-glucan diet and increased after β-glucan diet. These microbes had the ability to metabolize β-glucan or were beneficial to health. In conclusion, our findings demonstrate that variation in the composition of gut microbiota among individuals can result in varying expressions of β-glucan functionality. This outcome supports the notion that β-glucan may be metabolized through diverse pathways by gut microbes originally possessed by mice, subsequently producing various metabolites, such as short-chain fatty acids. Alternatively, the viscosity of the intestinal mucosa could be enhanced by β-glucan, potentially promoting the growth of certain bacteria (e.g., the genus *Akkermansia*). This study provides insights into the intricate interplay between β-glucan, gut microbiota, and immunomodulation.

## 1. Introduction

β-Glucan is a non-starch polysaccharide obtained from bacteria, algae, oats, and fungi [1]. It is recognized as a bioactive and functional food component, owing to its various biological effects, including modulation of the immune response, cholesterol reduction, blood sugar regulation, tumor suppression, and antioxidant activity. The β-glucan structure can vary depending on the source. For example, β-l,3- or β-l,4-glucan are found in barley and oats, and β-l,3- or β-l,6-glucan are major components of most fungi. However, most bacteria contain only β-l,3-glucan [2]. Depending on the structure of β-glucan, the immunomodulatory function appears differently [3]. β-glucan can stimulate immune cells by acting on multiple immune receptors such as Dectin-1, complement receptors 3 (CR3), and toll-like receptor (TLR)-2/6 [4,5]. This reaction induces the release of cytokines (e.g., IL-12, IL-6, IL-10, and TNF-α). The characteristics of β-glucan, such as its molecular weight, molecular structure (backbone or side chain), solubility, and particle size, can influence the immunomodulatory effects of β-glucan [6]. 

The COVID-19 pandemic has affected many industries and brought about changes in the market for health-functional food [7]. The health-functional food market is gradually expanding every year, and particularly due to this pandemic, there has been an increased interest in immunomodulatory functional foods [7,8]. Nevertheless, some customers have expressed dissatisfaction with health-functional foods, asserting that they did not perceive any advantages from using those products [9]. The effects of health-functional food can differ among individuals due to various reasons, and recent studies indicate that the composition of the gut microbiota plays a significant role in influencing the effects of health-functional foods on each individual [10,11]. The gut microbiota can influence the host’s metabolism and health by producing enzymes and synthesizing various metabolites [12]. Gut microbiota are recognized to have the potential to affect human health by participating in digestion, nutrient absorption, shaping the mucosal immune response, and the synthesis or modulation of many potentially bioactive compounds. The composition of gut microbiota can be easily reorganized by dietary exposure, resulting in individual variations. Even when consuming the same health-functional foods, the food can be metabolized differently by gut microbiota, leading to different responses among individuals [13].

Therefore, we hypothesized that the immunomodulatory effect of β-glucan can vary depending on the composition of an individual’s gut microbiota. To validate this, we administered β-glucan to mice *ad libitum*, classified them based on the degree of immunomodulatory effects, and compared the gut microbiota composition between the group with high immunomodulatory effect and the group with low immunomodulatory effect.

## 2. Materials and Methods

### 2.1. Experimental Design and Dosage Information

**Figure 1:**
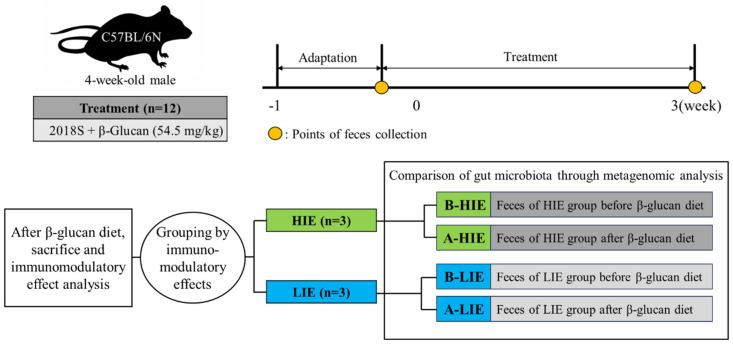
Experimental design. A-HIE, high immunomodulatory effect group after the diet of β-glucan; A-LIE, low immunomodulatory effect group after the diet of β-glucan; B-HIE, high immunomodulatory effect group before the diet of β-glucan; B-LIE, low immunomodulatory effect group before the diet of β-glucan; HIE, high immunomodulatory effect group; LIE, low immunomodulatory effect group.

Twelve male C57BL/6N mice (4-week-old, Laon Bio, Yongin, Republic of Korea) were housed under controlled conditions. For 3 weeks, the mice were fed 2018 Teklad global 18% protein rodent diets (Envigo, Indianapolis, IN, USA) containing 54.5 mg/kg β-glucan (Sigma-Aldrich, St. Louis, MO, USA) *ad libitum*. The concentration of dietary β-glucan was determined by considering the body weights of mice and humans, along with the permissible upper intake levels of β-glucan (163.4 μg per day) specified in the Ministry of Food and Drug Safety guidelines for the presence of functional components in health-functional foods [14]. Fresh feed was newly supplied on a weekly interval, and the intake of β-glucan was inferred by measuring the supply and residual amount of feed. On day 0 and day 21, fresh fecal samples were collected from each mouse; after fixing the mice, fresh feces were collected directly into 1.5 mL tubes from the anus by gently massaging the abdomen. After 3 weeks of diet, the mice were then sacrificed by overexposure to isoflurane inhalation [15]. Subsequently, the abdomen was incised to collect blood via the abdominal aorta vein, which was transferred to a serum separator tube. After allowing it to stand at room temperature for 20 min, the serum was isolated by centrifugation at 5000× *g* for 10 min at 4 °C. The serum was then transferred to a fresh 1.5 mL tube. Fecal samples, serum, and tissue samples were preserved at −70 °C until they were subjected to further analysis. In this study, the negative control (mice not fed a β-glucan diet) was not deeply examined; although an animal experiment was conducted for the control to visually assess the general health status of the mice, fecal analysis was not performed. Our focus was on evaluating the “high or low” immunomodulatory effect of β-glucan rather than determining its “presence or absence”. Thus, only the β-glucan feeding group was investigated. The overall schematic of the animal experiment is presented in Figure 1, and the Animal Experimental Ethics Committee of Sookmyung Women’s University approved all animal experiments (SMWU–IACUC–1912–023).

### 2.2. Classification into Groups with High and Low Immunomodulatory Effect

#### 2.2.1. Measurement of the Intake of β-Glucan and Confirmation of Mouse Health Status 

The dietary weights were measured weekly, including not only the food provided on top of the cage, but also any scattered food inside the cage, to determine the amount consumed by the mice during the week. This allowed verification of whether the intake of β-glucan was appropriate. Additionally, we conducted mouse body weight measurements and blood serum biochemical analysis to assess their overall health condition. A blood serum test was conducted for a total of nine markers, which include aspartate aminotransferase (AST), alanine aminotransferase (ALT), alkaline phosphatase (ALP), total protein, albumin, total bilirubin, blood urea nitrogen (BUN), creatine, and glucose.

#### 2.2.2. Analysis of Protein Expression Related to the Immunomodulatory Effects

To confirm the immunomodulatory effects of the β-glucan, total proteins were extracted from the mice’s colon, and western blotting was performed. To extract the total protein, 20 mg of colon tissue samples was homogenized in a 2 mL tube containing 600 μL of protein extraction solution (PRO-PREP^™^; iNtRON Biotechnology Inc., Seongnam, Republic of Korea) and stainless beads for 5 min at 50 Hz. Following beads removal, the homogenate was kept on ice for 30 min and subsequently centrifuged for 10 min at 11,463× *g* at 4 °C. The supernatant remaining after centrifugation was transferred to a fresh 2 mL tube, and it was regarded as containing the total protein content of the colon sample. The total protein concentration of each sample was measured using the DC^™^ Protein Assay (Bio-Rad Laboratories, Inc., Hercules, CA, USA) following the guidelines provided by the manufacturer. For size-based separation of total protein (40 μg), electrophoresis was conducted on a 12% Tris-glycine sodium dodecyl sulfate-polyacrylamide gel at 200 V for 30 min. The separated proteins were subsequently moved onto polyvinylidene difluoride membranes (GE Health Life Sciences, Marlborough, MA, USA) using a voltage of 60 V for 2.5 h. Following the transfer, the membranes were incubated with 5% skim milk at 25 °C for 1 h to block non-specific protein binding. Immunoblotting was performed at 4 °C for 12 h using primary antibodies against TNF-α (ab1793; Abcam, Cambridge, MA, USA), IL-6 (sc-57315), IL-10 (sc-365858), COX-2 (sc-376861), and iNOS (sc-7271) (Santa Cruz Biotechnologies, Inc., Dallas, TX, USA). As a reference, β-actin (sc-81178; Santa Cruz Biotechnologies, Inc.) was used. Anti-mouse IgG labeled with horseradish peroxidase (sc-2005; Santa Cruz Biotechnologies, Inc.) was used as the secondary antibody. The antigen/antibody complexes were visualized using ECL^™^ Select Western Blotting Detection Reagent (GE Health Life Sciences) for 1 min and then imaged using a LAS-3000 Imager (Fujifilm, Tokyo, Japan). The band intensity was quantified using GelQuant v.2.7. (DNR Imaging Systems Ltd., Jerusalem, Israel), and the relative expression levels of each protein were calculated based on the expression level of β-actin. Using these results, the mice were categorized into two groups: those showing high immunomodulatory effects of β-glucan (HIE) and those showing low immunomodulatory effects (LIE). Mice were assigned scores for the expression level of each protein, ranging from 1 (indicating the lowest immunomodulatory effect) to 12 (indicating the highest immunomodulatory effect), based on the result of the western blotting analysis. Subsequently, the scores for all markers were summed, and the three mice with high scores were classified into the HIE group, while the other three mice with low scores were classified into the LIE group.

### 2.3. Metagenomic Analysis

The composition of the gut microbiota in mice was determined using metagenomic analysis. The fecal samples were taken from the mice in the HIE and LIE groups before (named B-HIE and B-LIE, respectively) and after (named A-HIE and A-LIE, respectively) the administration of the β-glucan diet. DNA from fecal samples was extracted using DNeasy PowerSoil Kits (Qiagen, Hilden, Germany). The quantification of DNA was carried out using Quant-IT PicoGreen (Invitrogen, Carlsbad, CA, USA), following the provided instructions by the manufacturer. The sequencing library was prepared using the Illumina 16S metagenomic sequencing protocol. The DNA was amplified using 1 nM dNTP mix, 1X reaction buffer, 500 nM PCR primer, and 2.5 U of Herculase II fusion DNA polymerase (Agilent Technologies, Santa Clara, CA, USA), and purified using AMPure beads (Agencourt Bioscience, Beverly, MA, USA) as described by the manufacturer. Adapter and primer trimming were performed by Cutadapt. Sequencing of paired ends (2 × 300 bp) was conducted by the MiSeq^™^ platform (Illumina, San Diego, CA, USA). The sequence matching and attribution against databases were carried out using widely-used tools such as the BLAST, and the NCBI 16S database was utilized. The diversity and abundance of gut microbiota were invesigated by analyzing metagenomic sequences using the DADA2, and amplicon sequence variants (ASVs) for each group were then analyzed.

### 2.4. Statistical Analysis

SAS^®^ (v. 9.3; SAS Institute Inc., Cary, NC, USA) was used to perform statistical analysis of the data. A significant difference between groups was determined using a general linear model and pairwise *t*-tests at α = 0.05, followed by least-squares analysis.

## 3. Results and Discussion

### 3.1. β-Glucan Intake Does Not Affect Body Weight and Biochemical Indices

The mice were found to consume an average of 162.4 ± 18.1 μg of β-glucan per day, confirming that this amount is not significantly higher than our pre-established daily upper limit intake of 163.4 μg. The body weight of mice that were fed a β-glucan diet was an average of 15.8 ± 0.5 g at the beginning of the experiment to an average of 20.5 ± 1.5 g just before sacrifice. In the negative control, the average body weight of mice was 15.7 ± 0.3 g at the beginning, and 20.3 ± 1.1 g on average before sacrifice. It means that the increased level of weight observed is common, and based on this result, it is suggested that the consumption of β-glucan was appropriate. The serum biochemical markers, as presented in Table 1, were found to be within the normal range provided by Charles River [16]. AST and ALT are enzymes linked to liver and muscle health, and ALP is an enzyme found in the liver and bones. Total protein, including albumin, reflects nutritional and liver status. Total bilirubin associated with liver and bile duct function. BUN and creatine reveal kidney function, while glucose indicates metabolic health [17]. These markers are standard blood test parameters used to evaluate liver function, kidney function, protein levels, and glucose metabolism, offering essential insights into general health status and potential medical conditions [18]. Since all these biomarkers, which play such roles, were observed within the normal range, the health status of the mice was deemed to be without any issues.

### 3.2. The Immunomodulatory Effect of β-Glucan Was Observed Differently in Each Mouse

The immunomodulatory effect of β-glucan was analyzed by western blotting and assigned scores to each mouse range from 1 to 12 (Table 2; Supplementary Figure). The expression levels of COX-2 were observed to be highest in mouse No. 11 and the lowest in mouse No. 1. iNOS expression showed the highest level in mouse No. 12 and the lowest level in mouse No. 1. In terms of IL-6 expression, mouse No. 3 exhibited the highest level, while mouse No. 9 had the lowest level. On the other hand, for the anti-inflammatory cytokine IL-10, mouse No. 4 showed the highest expression, while mouse No. 12 displayed the lowest expression. The scores for the indicators were summed for each mouse, leading to their categorization into either the HIE group (including mice No. 7, No. 11, and No. 12) or the LIE group (including mice No. 2, No. 9, and No. 10). Afterward, statistical analysis between the HIE and LIE groups was conducted to compare the expression levels of each protein. The HIE group exhibited significantly elevated expression levels of COX-2 and iNOS compared to the LIE group (*p* < 0.05; Table 3). Studies have demonstrated that β-glucan enhances macrophage function and boosts the antimicrobial activity of neutrophils and mononuclear cells. The production of pro-inflammatory cytokines and chemokines is also elevated, resulting in an improved immune response [19,20]. Smiderle et al. (2011) identified an increase in *TNF-α* and *COX-2* genes expression after treating human monocytic THP-1 cells with polysaccharide extracts from mushrooms [21]. TNF-α plays a crucial role in regulating inflammatory responses and activating mediators at the terminal end of the cytokine cascade. It also serves as a potent inducer of other pro-inflammatory cytokines. This pivotal role makes TNF-α a central player in orchestrating inflammation and immune activation [22]. Furthermore, in ovine ruminal explants, β-glucan was found to stimulate IL-6 and IL-10 production, which supports mucosal immunity [23]. IL-10, which primarily exerts anti-inflammatory effects, significantly inhibits dendritic cell activation, and it suppresses the production and secretion of inflammatory cytokines from dendritic cells. IL-10 has been identified as a cytokine that functions as an inhibitor of cytokine synthesis, as it can suppress the production of TNF-α, IL-1, IL-6, and IL-8 [22]. Therefore, we conducted our study based on the assumption of an inverse correlation between IL-10 and immune-enhancing effects. Under normal physiological conditions, iNOS enzymatically transforms L-arginine into L-citrulline, leading to the production of NO within cells [24]. NO helps defend against bacterial, fungal, and parasitic infections as a macrophage effector. Heightened iNOS expression can lead to the production of NO, which may exert diverse impacts on immune cells. COX-2 creates an immunosuppressive network and triggers the transcription factor FoxP3, which increases Treg cell activity [25]. Even though the mice in the HIE and LIE groups consumed the same amount of β-glucan, the immunomodulatory effect was different in both groups. Thus, we hypothesized that this difference in immunomodulatory effect between the two groups of mice could be attributed to variations in their gut microbiota, despite providing them with the same diet and same environment. To test this hypothesis, we conducted the metagenomic analysis using fecal samples obtained from both groups of mice on day 0 (before β-glucan diet; B-HIE and B-LIE) and day 21 (after β-glucan diet; A-HIE and A-LIE) of the animal experiment.

### 3.3. The Immunomodulatory Effect of β-Glucan Depends on the Gut Microbiota

**Figure 2:**
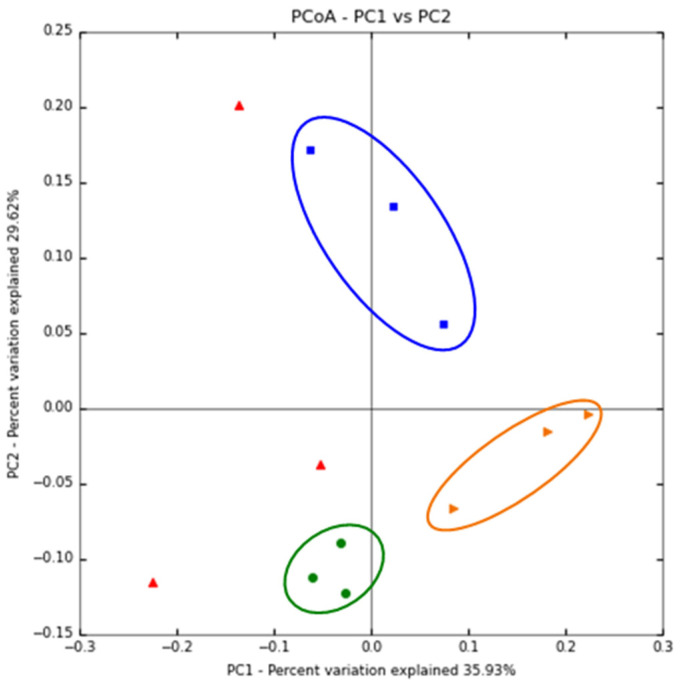
Principal coordinate analysis (PCoA) plot of all groups. A-HIE (red-triangle), high immunomodulatory effect group after the diet of β-glucan; A-LIE (blue-square), low immunomodulatory effect group after the diet of β-glucan; B-HIE (orange-triangle), high immunomodulatory effect group before the diet of β-glucan; B-LIE (green-circle), low immunomodulatory effect group before the diet of β-glucan.

**Figure 3:**
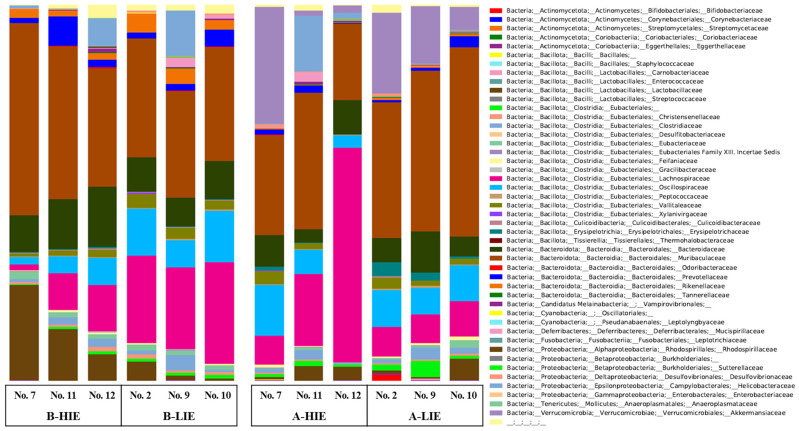
Bar charts of relative abundance ratio at family level in gut microbiota of mice feces. B-HIE, high immunomodulatory effect group before the diet of β-glucan; B-LIE, low immunomodulatory effect group before the diet of β-glucan; A-HIE, high immunomodulatory effect group after the diet of β-glucan; A-LIE, low immunomodulatory effect group after the diet of β-glucan.

The Shannon index is utilized to describe the diversity (richness and evenness) of the microbial populations within groups [26]. Before feeding a β-glucan diet, the Shannon index was observed to be 6.1 ± 0.4 for the B-HIE group and 6.7 ± 0.3 for the B-LIE group. After feeding, it decreased to 5.3 ± 1.1 for the A-HIE group and 6.2 ± 0.1 for the A-LIE group, confirming the reduction in diversity. The Shannon index is calculated by considering not only the number of species but also the distribution of bacteria within each species [27]. Thus, a decrease in diversity means that a specific microorganism has become dominant, and in the study of Myhrstad et al. (2020), the diversity was also reduced due to the β-glucan diet [28]. In other words, the consumption of β-glucan can lead to an increase in the dominance of specific microbial species over others, resulting in a decrease in diversity. A principal coordinate analysis (PCoA) plot was utilized to visualize the diversity of gut microbiota. We found that the B-LIE, B-HIE, and A-LIE groups were completely divided (Figure 2), which means that the distinct separation of each group implies different composition of the gut microbiota between them. The PCoA plot represents the data in a reduced dimensional space, allowing researchers to observe patterns of similarity and dissimilarity between the groups based on their gut microbial composition, emphasize major microbial trends. In the PCoA plot, every data point corresponds to a sample, and the proximity of the points indicates the level of similarity between the gut microbiota of those samples [29,30]. The B-LIE, B-HIE, and A-LIE groups showed similar dominant microbial species or clusters within their mouse, suggesting that the individuals within each group share a similar microbial composition. This observation is supported by the finding in Figure 3. In contrast, the A-HIE group exhibited varying microbial compositions among individuals, with different dominant microbial species. As a result, the distance between data points in the plot appears more significant. Based on these results, we can tentatively speculate that the expression level of β-glucan effect is influenced by the baseline gut microbiota composition in mice. However, PCoA may emphasize the composition of principal microbial populations, making it challenging to discern subtle differences. Therefore, it is important to complement PCoA with consideration of tzxonomic abundance to fully capture subtle variations.

**Figure 4:**
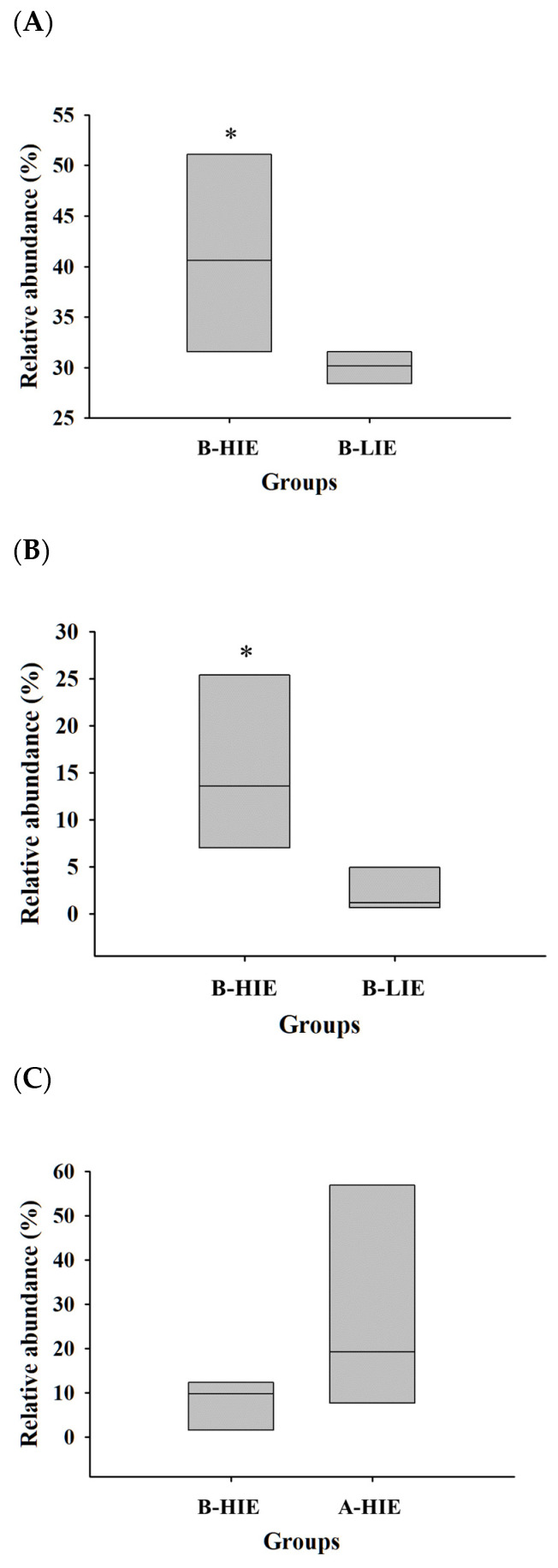
Relative abundance ratio (%) of the family *Muribaculaceae* (**A**), family *Lactobacillaceae* (**B**), and family *Lachnospiraceae* (**C**). B-HIE, high immunomodulatory effect group before the diet of β-glucan; B-LIE, low immunomodulatory effect group before the diet of β-glucan; A-HIE, high immunomodulatory effect group after the diet of β-glucan. * Statistically significant, compared two groups by paired *t*-test (*p* < 0.05).

The taxonomic composition of each group was studied to better understand the microbial community. The proportion of the phylum Bacteroidota (synonym Bacteroidetes) was significantly higher in the B-HIE group (59.96 ± 4.11%) than in the B-LIE group (45.75 ± 1.79%) (Table 4; *p* < 0.05). Also, the phylum Verrucomicrobia was absent before the β-glucan diet, but it was observed to be present in more than 10% after the β-glucan diet (Table 4). At the family level, the main bacteria were of the family *Muribaculaceae*, and their proportion was 41.08 ± 5.64% in the B-HIE group and 30.03 ± 0.91% in B-LIE (Figure 3 and Figure 4A; *p* < 0.05). The proportion of the family *Lactobacillaceae* was significantly observed to be high in B-HIE (15.31 ± 5.37%) rather than in B-LIE (2.25 ± 1.36%) (Figure 3 and Figure 4B; *p* < 0.05). Also, the family *Lachnospiraceae* tended to increase with the diet of β-glucan in the group with a high immunomodulatory effect of β-glucan; the proportions were 7.87 ± 3.24% in the B-HIE group and 27.91 ± 14.88% in the A-HIE group (Figure 3 and Figure 4C). Several prior studies, investigating the ingestion of β-glucan, have similarly observed an increase in phylum Bacteroidota, genus *Lactobacillus*, and genus *Akkermansia* abundance, consistent with our study findings [31,32,33].

For β-glucan to have the immunomodulatory effect, it must contain a β-1,3 glycosidic bonds chain with a length of at least seven glucose units and β-1,6 glycosidic bonds in the side chain [3]. It cannot be degraded by the mammalian digestive system and binds to the macrophage membrane receptors Dectin-1, an integral membrane protein of type II with a carbohydrate recognition domain akin to a C-type lectin, and TLR-2/6, a heterodimeric receptor complex involved in recognizing specific molecular patterns on pathogens and activating immune responses [34,35]. It is then introduced into the cell by endosomes and fragmented. Different fragments attach to the CR3 membrane receptors of mononuclear cells, macrophages, dendritic cells, and natural killer cells, inducing the secretion of TNF-α and various cytokines. This results in stimulation of both innate and adaptive immunity [36]. Importantly, β-glucan can be broken down by the carbohydrate-degrading enzymes produced by certain species in the gut microbiota. The phylum Bacteroidota plays a role as glycan degraders by producing enzymes such as glycoside hydrolases and polysaccharide lyases [37]. Thus, it is speculated that in the guts of mice with a higher abundance of them, the metabolism of β-glucan was more actively promoted, leading to a higher expression of its immunomodulatory effects. The family *Muribaculaceae* are abundant bacteria in the murine gut and have been reported to have a positive correlation with propionate, known as a beneficial short-chain fatty acid (SCFA) [38,39,40]. Wang et al. (2022) revealed that β-glucan regulates host immunity by elevating the family *Lachnospiraceae* [41]. The family *Lachnospiraceae* is known to produce SCFA, especially butyrate. SCFA has important roles in providing energy to colonocytes and modulating immune responses. They can influence the recruitment of neutrophils to different tissues. This is accomplished by controlling the production of inflammatory mediators, which include cytokines such as TNF-α and IL-17, along with neutrophil-chemoattractants [42]. Butyrate plays a crucial role in promoting the differentiation of regulatory T cells through interactions with specific G protein-coupled receptors on dendritic cells and CD4+ T cells [43]. We identified 4 genera belonging to the family *Lactobacillaceae*, namely, *Lactobacillus*, *Ligilactobacillus*, *Limosilactobacillus*, and *Liquorilactobacillus*. They are known to have immunomodulatory effects on their own. For example, *Limosilactobacillus reuteri* produces exopolysaccharide (EPS) to regulate the immune response [44]. EPS is a complex carbohydrate produced and released by bacteria into their external environment. It has been shown to play a crucial role in modulating the immune response and supporting gut health. EPS can interact with immune cells, such as dendritic cells and T cells, promoting anti-inflammatory responses while inhibiting pro-inflammatory signals. This modulation contributes to a more balanced and appropriate immune reaction, potentially reducing the risk of inflammatory conditions [45]. Furthermore, EPS may also be involved in creating a protective mucosal barrier in the gut. This barrier helps maintain the integrity of the intestinal lining and prevents the entry of harmful pathogens and toxins into the bloodstream [46]. These results suggest that β-glucan’s immunomodulatory effect may be more active depending on the composition of gut microbiota that mice originally had in the gut. Only the genus *Akkermansia* in the phylum Verrucomicrobia was observed, and it is known as a representative mucin-degrading bacterium that positively impacts the host’s health [47,48]. β-Glucan is one of the dietary fibers such as cellulose, gum, and pectin [49,50]. Dietary fibers are not broken down by digestive enzymes and instead promote the production of mucins, increasing the viscosity of the intestines [50,51,52]. The viscosity caused by β-glucan can alter the composition and activity of the gut microbial community. In general, the abundance of the genus *Akkermansia* is upregulated with dietary fiber intervention by providing mucin as a substrate [52,53]. Ryan et al. (2017) showed that the genus *Akkermansia* population was elevated following the intake of β-glucan, as in our research [54]. In the gut, various bacteria inhabit in the mucus membrane, and the genus *Akkermansia* also inhabits in the membrane [47,55,56]. From a different point of view, if the colonic mucosa is shed, the genus *Akkermansia* may leak out along with the colonic mucosa and be detected in feces. Shedding of the colonic mucosa usually occurs when the tight junction within the colonic mucosa is weakened, which is a representative symptom of leaky gut syndrome [57]. However, it was confirmed that the genus *Akkermansia* enhances the integrity of the colon by strengthening the tight junction of the colonic mucosa [57,58]. Therefore, the increase in the genus *Akkermansia* in feces identified in this study is not due to the outflow of colonic mucosa. This change occurred solely due to the consumption of β-glucan and can be attributed to a positive impact on the host’s health. Additionally, the genus *Akkermansia* is also a bacterium with the ability to produce SCFAs as metabolic by-products [59]. The genus *Akkermansia* can contribute to the immune response by inducing IgG1 production and specific T-cell-related responses, and it appears to trigger T-cell responses independently via follicular T-cells. This interaction between T-cell-dependent responses and the microbiota, particularly the genus *Akkermansia* seems to enhance host immunity as part of the homeostasis process [60]. As a result, β-glucan may have improved the gut microbiota apart from its immunomodulatory effects. Since actual metabolic product experiments were not conducted in this study, it cannot be definitively confirmed that metabolites such as SCFA were produced due to gut microbiota. However, several previous studies have already demonstrated that the functionality of specific substances can be altered by metabolites produced by gut microbiota. Therefore, it is plausible to consider that the immunomodulatory effect of β-glucan could be influenced by metabolites generated by gut microbiota, although this remains a “possibility”.

## 4. Conclusions

The impact of β-glucan on immunomodulation can be contingent upon the composition of the host’s gut microbiota. If mice have a high abundance of specific microbes (e.g., phylum Bacteroidota and family *Muribaculaceae*), the metabolism of β-glucan by these gut microbes may become more active, leading to elevated immunomodulatory effects. Additionally, the β-glucan diet may promote the growth of certain gut microbes (e.g., family *Lachnospiraceae* and genus *Akkermansia*) that were initially present in very low proportion, thereby improving the immunomodulatory effect. As revealed in this study, individual variations in gut microbiota composition may lead to different expressions of β-glucan functionality in individuals. Some individuals may have specific microbial species or clusters in their gut that are particularly efficient in metabolizing β-glucan, resulting in higher immunomodulatory effects. On the other hand, others may lack such microbial species or have different dominant species, leading to lower immunomodulatory effects. Furthermore, β-glucan intake may influence the abundance of certain gut microbes (e.g., the genus *Akkermansia*) in the intestinal mucosa, potentially contributing positively to health. Ultimately, the properly regulated gut microbiota by β-glucan can enhance the effects of β-glucan. Given the increased interest in health due to the pandemic, it is foreseeable that the development of suitable supplements through future research would hold significant value in improving health outcomes. Additional investigations in this field have the potential to reveal distinct microbial patterns that are associated with improved β-glucan functionality. This could lead to the development of personalized interventions and precision nutrition approaches, tailored to individuals according to their unique gut microbiota composition.

## Figures and Tables

**Table 1:** Blood serum biochemical analysis.

Markers	Concentration
AST	52.6 ± 3.3 U/L
ALT	26 ± 1.2 U/L
ALP	147 ± 4.5 U/L
Total protein	5.4 ± 0.1 g/dL
Albumin	3.4 ± 0.0 g/dL
Total bilirubin	0.5 ± 0.1 mg/dL
BUN	23.3 ± 1.0 mg/dL
Creatine	0.4 ± 0.0 mg/dL
Glucose	157 ± 8.7 mg/dL

AST, aspartate aminotransferase; ALT, alanine aminotransferase; ALP, alkaline phosphatase; BUN, blood urea nitrogen. Data expressed as mean ± standard error.

**Table 2:** Expression levels of proteins related to immunomodulatory effect of β-glucan in colon tissue and scores based on their expression levels.

Mouse Object No.	COX-2		IL-6		IL-10		iNOS		TNF-α		Total Score
	Value	Score *	Value	Score	Value	Score	Value	Score	Value	Score	
1	0.087	1	0.655	11	0.131	11	0.138	1	0.120	2	26
2	0.102	2	0.529	8	0.233	7	0.200	3	0.168	6	26
3	0.151	6	0.773	12	0.267	5	0.240	4	0.170	7	34
4	0.193	9	0.388	5	0.349	1	0.308	6	0.299	12	33
5	0.170	7	0.449	7	0.286	4	0.258	5	0.231	11	34
6	0.111	3	0.602	10	0.147	10	0.165	2	0.151	4	29
7	0.307	11	0.545	9	0.221	8	0.529	10	0.162	5	43
8	0.182	8	0.369	4	0.306	2	0.462	9	0.182	8	31
9	0.118	4	0.228	1	0.300	3	0.395	7	0.196	9	24
10	0.144	5	0.410	6	0.254	6	0.424	8	0.114	1	26
11	0.378	12	0.332	2	0.187	9	0.532	11	0.217	10	44
12	0.227	10	0.365	3	0.110	12	0.648	12	0.133	3	40

COX-2, cyclooxygenase-2; IL-6, interleukin-6; IL-10, interleukin-10; iNOS, inducible nitric oxide synthase; TNF-α, tumor necrosis factor-α. * The immunomodulatory effects were evaluated and scored on a scale of 1 to 12, with higher scores indicating a higher immunomodulatory effect. Scores for all indicators were then summed and the mice were divided into two groups based on the total scores.

**Table 3:** Comparison protein expression levels of high immunomodulatory effect (HIE) group and low immunomodulatory effect (LIE) group after the β-glucan diet.

Protein	HIE	LIE
COX-2/β-actin	0.30 ± 0.04	0.12 ± 0.01 *
IL-6/β-actin	0.41 ± 0.07	0.39 ± 0.09
IL-10/β-actin	0.17 ± 0.03	0.26 ± 0.02
iNOS/β-actin	0.57 ± 0.04	0.34 ± 0.07 *
TNF-α/β-actin	0.17 ± 0.02	0.16 ± 0.02

HIE, high immunomodulatory effect group (three mice with the highest total scores); LIE, low immunomodulatory effect group (three mice with the lowest total scores); COX-2, cyclooxygenase-2; IL-6, interleukin-6; IL-10, interleukin-10; iNOS, inducible nitric oxide synthase; TNF-α, tumor necrosis factor-α. Data expressed as mean ± standard error. * Statistically significant, compared two groups by paired *t*-test (*p* < 0.05).

**Table 4:** Relative abundance ratio (%) in fecal microbiota at phylum level.

Phylum	Groups			
	B-HIE	B-LIE	A-HIE	A-LIE
Actinomycetota	0.09 ± 0.01	0.07 ± 0.02	0.16 ± 0.07	0.73 ± 0.56
Bacillota (Synonym Firmicutes)	34.82 ± 0.39	46.42 ± 2.85	44.03 ± 10.85	30.33 ± 1.31
Bacteroidota (Synonym Bacteroidetes)	59.96 ± 4.11 ^a^	45.75 ± 1.79 ^b^	36.34 ± 3.52 ^c^	52.68 ± 4.59 ^a^
Candidatus Melainabacteria	0.38 ± 0.32	0.22 ± 0.07	0.26 ± 0.17	0.08 ± 0.05
Cyanobacteria	0.03 ± 0.03	0.04 ± 0.02	- *	-
Deferribacteres	0.03 ± 0.02	1.29 ± 0.73	0.98 ± 0.83	0.07 ± 0.03
Fusobacteria	-	0.002 ± 0.002	-	-
Proteobacteria	3.03 ± 2.46	4.39 ± 4.05	6.00 ± 4.54	0.80 ± 0.14
Tenericutes	0.10 ± 0.06	0.03 ± 0.02	0.05 ± 0.03	0.01 ± 0.01
Verrucomicrobia	-	-	11.46 ± 9.83	14.23 ± 4.50
Uncultured	1.56 ± 0.96	1.79 ± 0.28	0.73 ± 0.39	1.06 ± 0.55

B-HIE, high immunomodulatory effect group before the diet of β-glucan; B-LIE, low immunomodulatory effect group before the diet of β-glucan; A-HIE, high immunomodulatory effect group after the diet of β-glucan; A-LIE, low immunomodulatory effect group after the diet of β-glucan. Data expressed as mean ± standard error. ^(a–c)^ Different letters in a same row indicate statistically significant *p*-value (*p* < 0.05). * not found in fecal microbiota.

## Data Availability

The data used to support the findings of this study can be made available by the corresponding author upon request.

## Supplementary Materials

The following supporting information can be downloaded at: https://www.mdpi.com/article/10.3390/foods12173148/s1, Figure S1: The gel images and expression level of proteins related with immune response in each mouse.

## Abbreviations

ALP, alkaline phosphatase; ALT, alanine aminotransferase; AST, aspartate aminotransferase; ASV, amplicon sequence variants; A-HIE, high immunomodulatory effect after the β-glucan diet; A-LIE, low immunomodulatory effect after the β-glucan diet; BUN, blood urea nitrogen; B-HIE, high immunomodulatory effect before the β-glucan diet; B-LIE, low immunomodulatory effect before the β-glucan diet; COX, cyclooxygenase; EPS, exopolysaccharide; HIE, high immunomodulatory effect; IL, interleukin; iNOS, inducible nitric oxide synthase; LIE, low immunomodulatory effect; PCoA, Principal coordinate analysis; SCFA, short-chain fatty acid; TNF, tumor necrosis factor.
