# Supplementary material for: The Immunomodulatory Effect of β-Glucan Depends on the Composition of the Gut Microbiota
Source: Foods. 2023 Aug 22;12(17):3148. doi: 10.3390/foods12173148 (PMC10487241; doi:10.3390/foods12173148)
Supplement: Supplementary file 1 [file foods-12-03148-s001.zip › foods-2534721-supplementary.pdf]

Supplementary Figure

A. COX–2

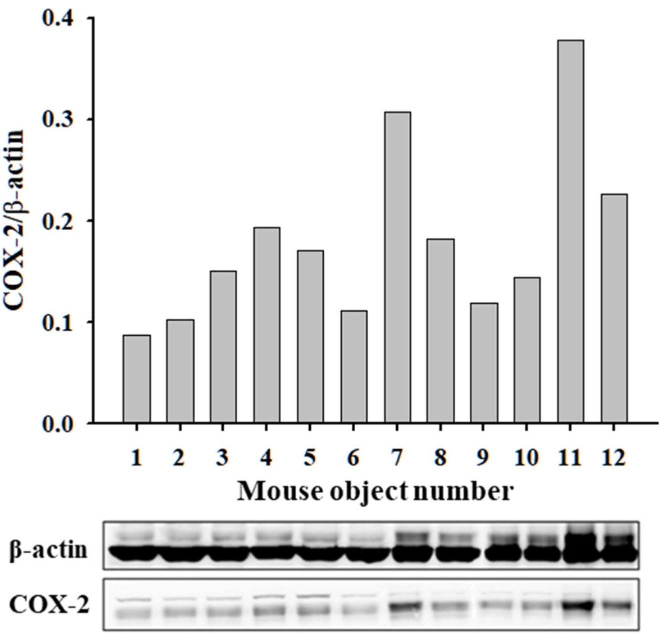

B. IL–6

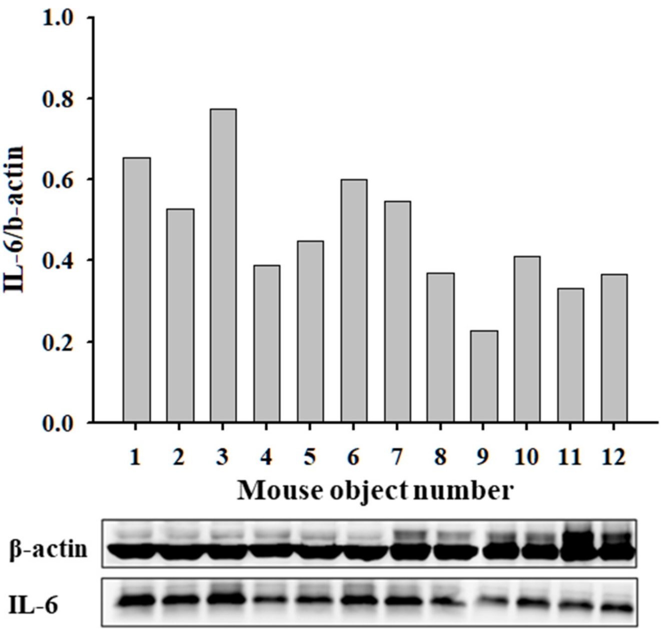

C. IL–10

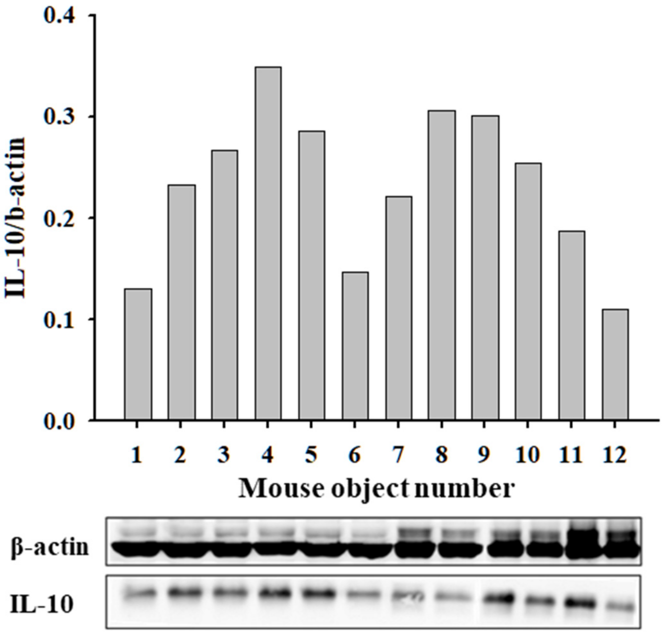

0.4
0.3
0.2
0.1
0.0
IL-10/b-actin
1 2 3 4 5 6 7 8 9 10 11 12
Mouse object number
β-actin
IL-10

D. iNOS

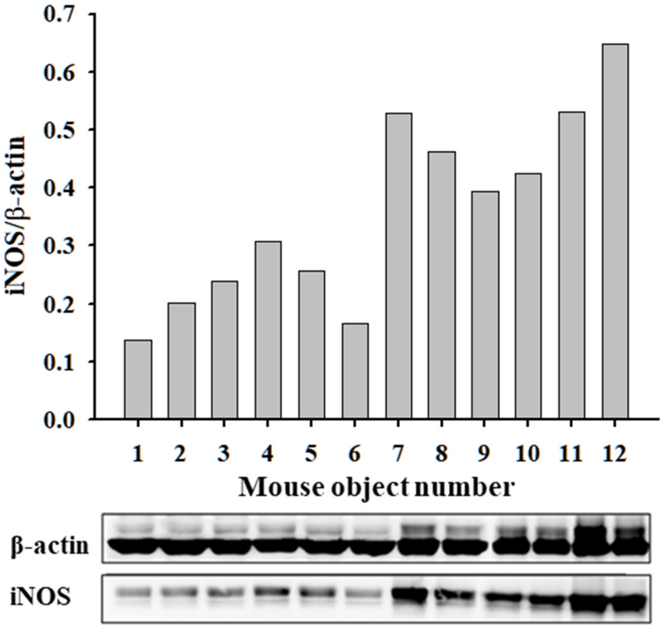

0.7
0.6
0.5
0.4
0.3
0.2
0.1
0.0
iNOS/β-actin
1 2 3 4 5 6 7 8 9 10 11 12
Mouse object number
β-actin
iNOS

E. TNF–α

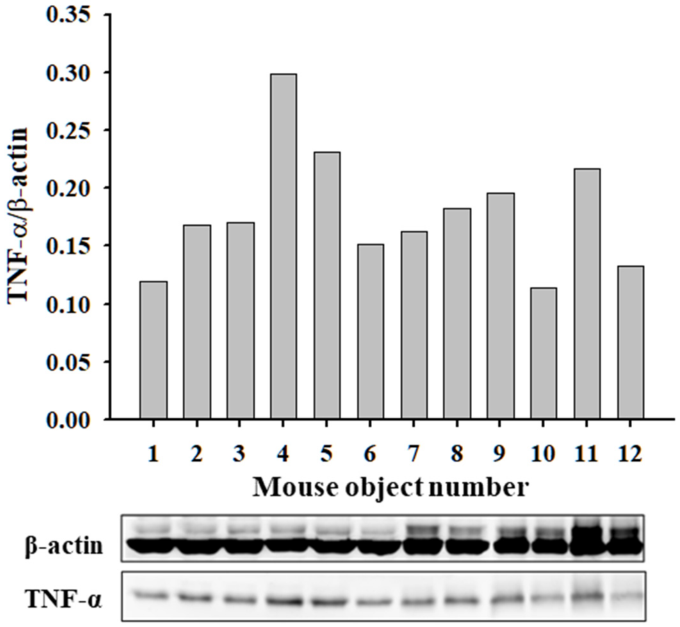

Supplementary Figure S1. The gel images and expression level of proteins related with immune response in each mouse.
